# Representational risks associated with interview-based animated documentaries

**DOI:** 10.1386/ap3_00032_1

**Published:** 2023-12-20

**Authors:** Alex Widdowson

**Affiliations:** Queen Mary University of London

**Keywords:** ethics, autism, evocation, non-mimetic substitution, gaze, Othering, unconscious bias, practice-based research

## Abstract

This article provides an analysis of representational issues associated with interview-based animated documentary productions directed by non-autistic filmmakers, attempting to represent one or more autistic participants. The article draws insights from three case studies: A Is for Autism *(Webb 1992)*, An Alien in the Playground (Glynne 2009) and the author’s own practice-based research film, Drawing on Autism (Widdowson 2021). Drawing insights from psychoanalysis, film theory and ethnography, the article will examine animated documentary practice in terms of the risks of Othering participants, look for evidence of the filmmaker’s unconscious bias and consider how the cinematic gaze can be used to decode ideological systems that informed their construction. From this analysis, the author reaches the conclusion that the properties of documentaries, that rely on animation rather than live-action cinematography, present a specific set of ethical responsibilities that skew towards issues of representation.

## Introduction

This article will provide an analysis of representational issues associated with interview-based animated documentary productions that employ non-mimetic substitution and evocation techniques. I have devised a comparative study of three animated documentaries that address the topic of autism – *A Is for Autism* (Webb 1992), *An Alien in the Playground* (Glynne 2009) and my own film, *Drawing on Autism* (Widdowson 2021), which builds upon insights drawn from the previous examples. My primary focus is how the properties of documentaries that rely on animation, rather than live-action cinematography, present a specific set of ethical responsibilities that skew towards issues of representation. The three case studies are all examples of animated documentaries directed by non-autistic filmmakers attempting to represent one or more autistic documentary participants.^1^ Drawing insights from psychoanalysis, film theory and ethnography, I will examine how these films, including my own, navigate the risk of Othering their participants, look for evidence of the film-maker’s unconscious bias and consider how the cinematic gaze can be used to decode ideological systems that informed their construction. Finally, I will explain how this research approach shaped my own practice-based research when creating *Drawing on Autism*.

## Autism in animated documentaries: Three case studies

**Figure 1:**
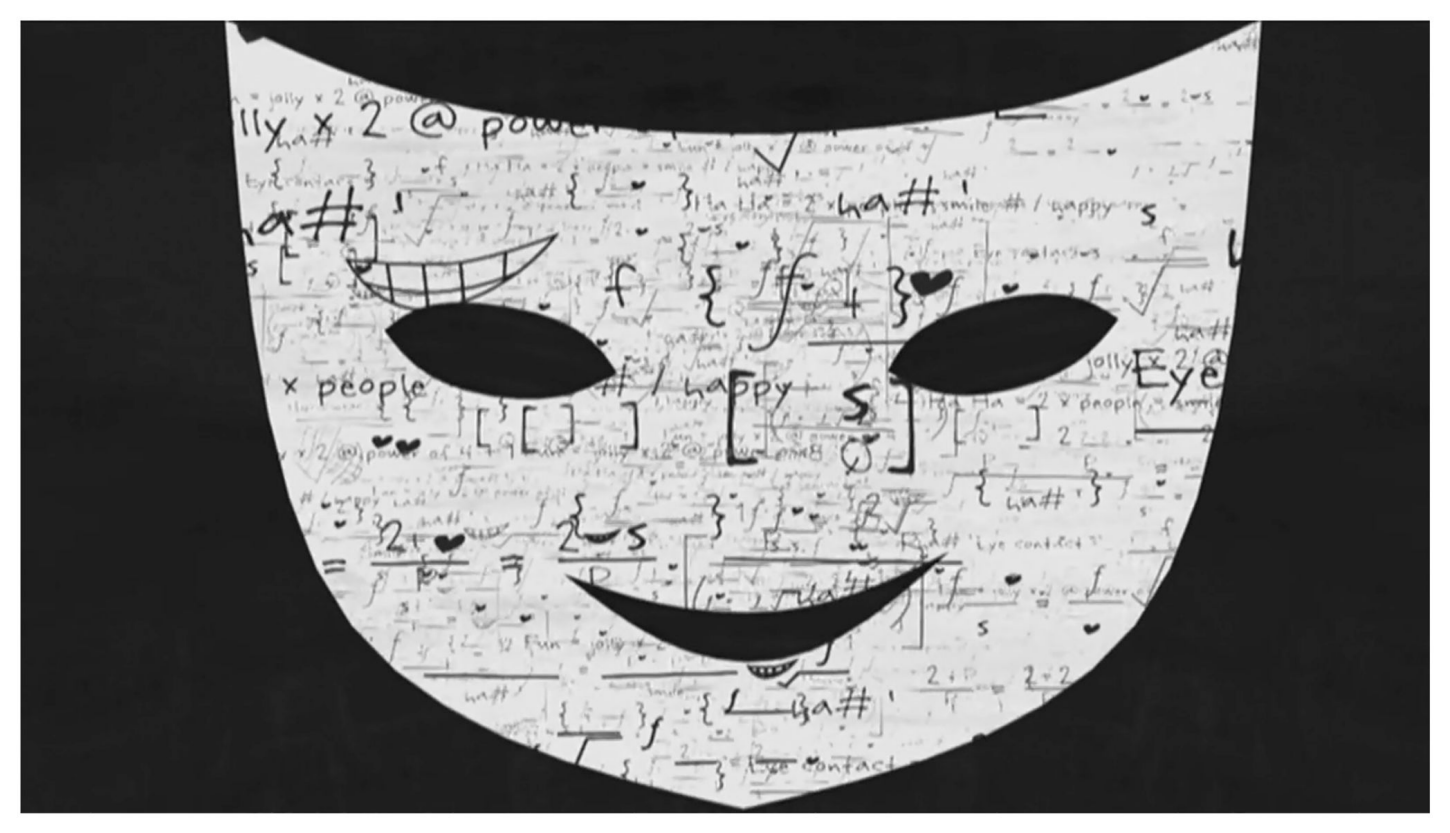
*Still from* An Alien in the Playground, *A. Glynne (dir.), 2009. UK: Channel 4. © Mosaic Films*.

*An Alien in the Playground* (2009) was directed by Andy Glynne. This short film is part of a miniseries, *Animated Minds* (Glynne 2003–09). In each episode, a single documentary participant gives a first-hand account that describes their form of neurological or psychological difference. The episode titled ‘An Alien in the Playground’ features the autobiographical narration of Joshua Muggleton, a teenager with^2^ Asperger’s syndrome,^3^ describing the anxiety he experienced during his education. He refers to his disinterest in social play at primary school, his lack of aptitude for non-verbal communication and experiences being bullied in secondary school. Muggleton links sensory over-stimulation in the classroom to poor concentration and emotional stress, and he describes a system of social strategies he consciously formulated for each person he knew in order to navigate social interactions. The animation frequently illustrates key phrases or words from the testimony, so when Muggleton compares using his social strategies to wearing a mask, the inverted silhouette of a Thalia theatre mask appears, framing the existing composition (Figure 1). At other times, connections between the testimony and aesthetic choices are less clear. When Muggleton describes navigating secondary school, his character avatar is depicted climbing ladders that lead up textured angular shapes, as abusive phrases appear letter by letter from the mouths of large brightly coloured heads. Muggleton’s character avatar is mimetic in that it is recognizably human and matches the narrator’s pale skin and dark spiky hair, but it would be hard to identify him based on the design. However, Muggleton is credited, so cannot be considered fully anonymized. Matthew Morgan wasthe animation director of this episode; however, Andy Glynne is credited as the producer and director of the series. It is unclear which creative decisions were Glynne’s and which were Morgan’s. Glynne explains that some of the animators responsible for *Animated Minds* episodes required little direction, while he felt others needed detailed instructions (Glynne 2012: 298). For simplicity, in this article, the aggregated work of each production team will be attributed to a single auteur filmmaker, in this case, Andy Glynne. While Glynne is not himself autistic, his work is informed by his experience as a clinical psychologist. Dr Joshua Muggleton, aged 32 at the time of writing, also went on to qualify as a clinical psychologist (Muggleton n.d.: n.pag.).

**Figure 2:**
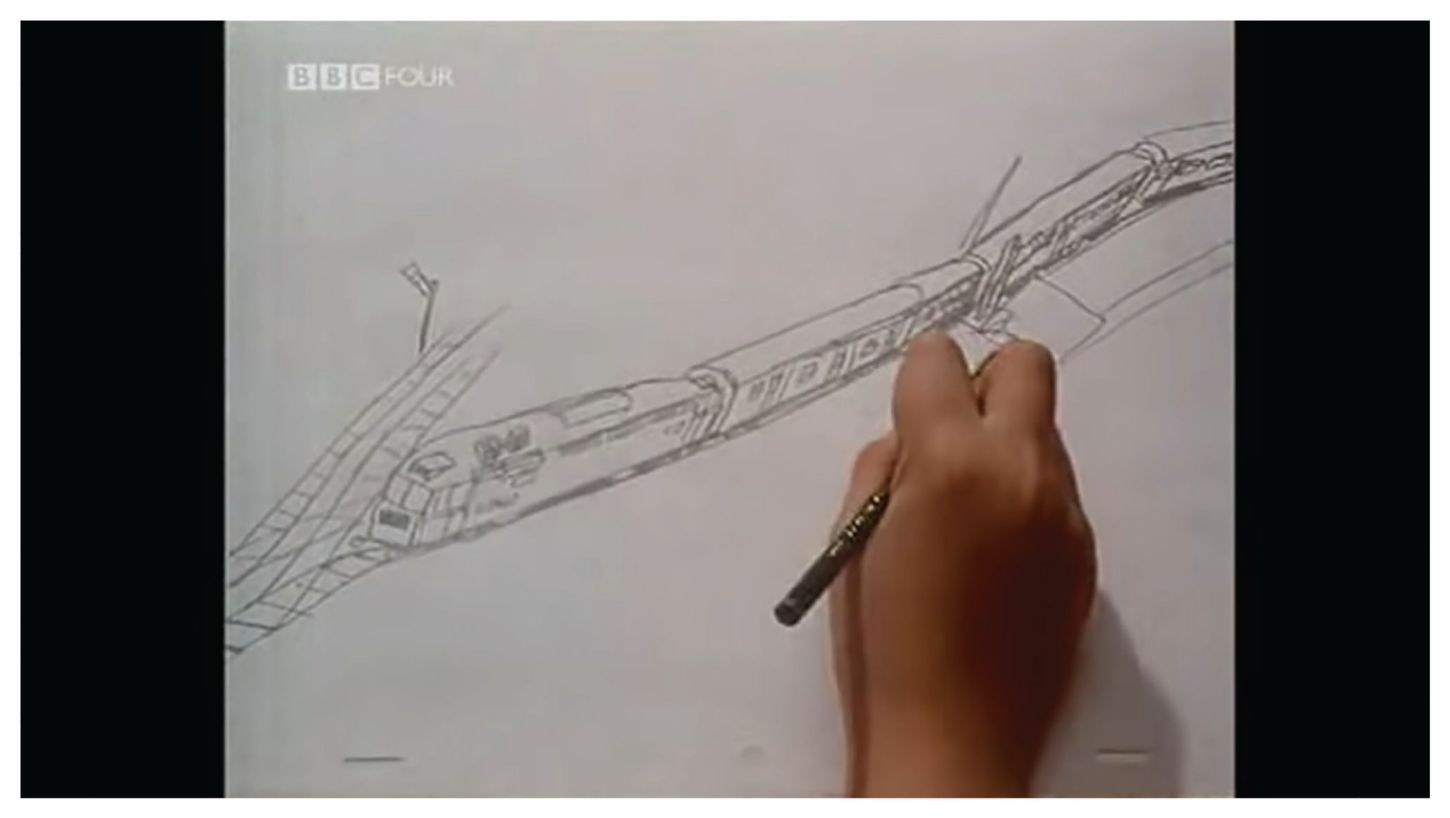
*Still from* A Is for Autism, *T. Webb (dir.), 1992. UK: Channel 4. © Channel 4*.

*A Is for Autism* (1992) was directed by Tim Webb. This hybrid animated documentary short includes some live-action elements. The art direction of the 2D animated scenes was based on the drawings of autistic artists and the narrative was organized around the first-person testimony of seven autistic narrators, one of whom had their words performed by an actor. Like *An Alien in the Playground*, the film is focused on the task of explaining what it is like being autistic, primarily for the benefit of non-autistic audiences. The topics addressed include special interests, struggles socializing, difficulty developing speech and literacy, hypo/hyper-sensory sensitivity and trouble picking up on non-verbal cues. While the animation was mostly completed by two non-autistic animators – Tim Webb and Ron MacRae – Daniel Sellers, an autistic child, designed, animated and narrated scenes that demonstrated his passion for trains (Figure 2). All other animated sequences were based on one or more artworks created by autistic contributors. The correlation between the art direction of each scene and the autistic narrators is inconsistent. For example, Temple Grandin, one of the earliest and best-known autistic autobiographical writers, did not create the artworks that illustrate her statements. Likewise, hundreds of credited and uncredited artworks, some collected from specialist schools and charities, are incorporated into the film accompanying unconnected testimony. The film consists of a complex tapestry of collaboration between Webb, MacRae and many autistic contributors. Webb attributed the idea of organizing the production around the principle of collaboration to Claire Kitson, the then commissioning editor of Channel 4, the film’s funder (Moore 2015: 51). This guiding principle is foregrounded by the film’s subtitle, ‘a collaboration’.

**Figure 3:**
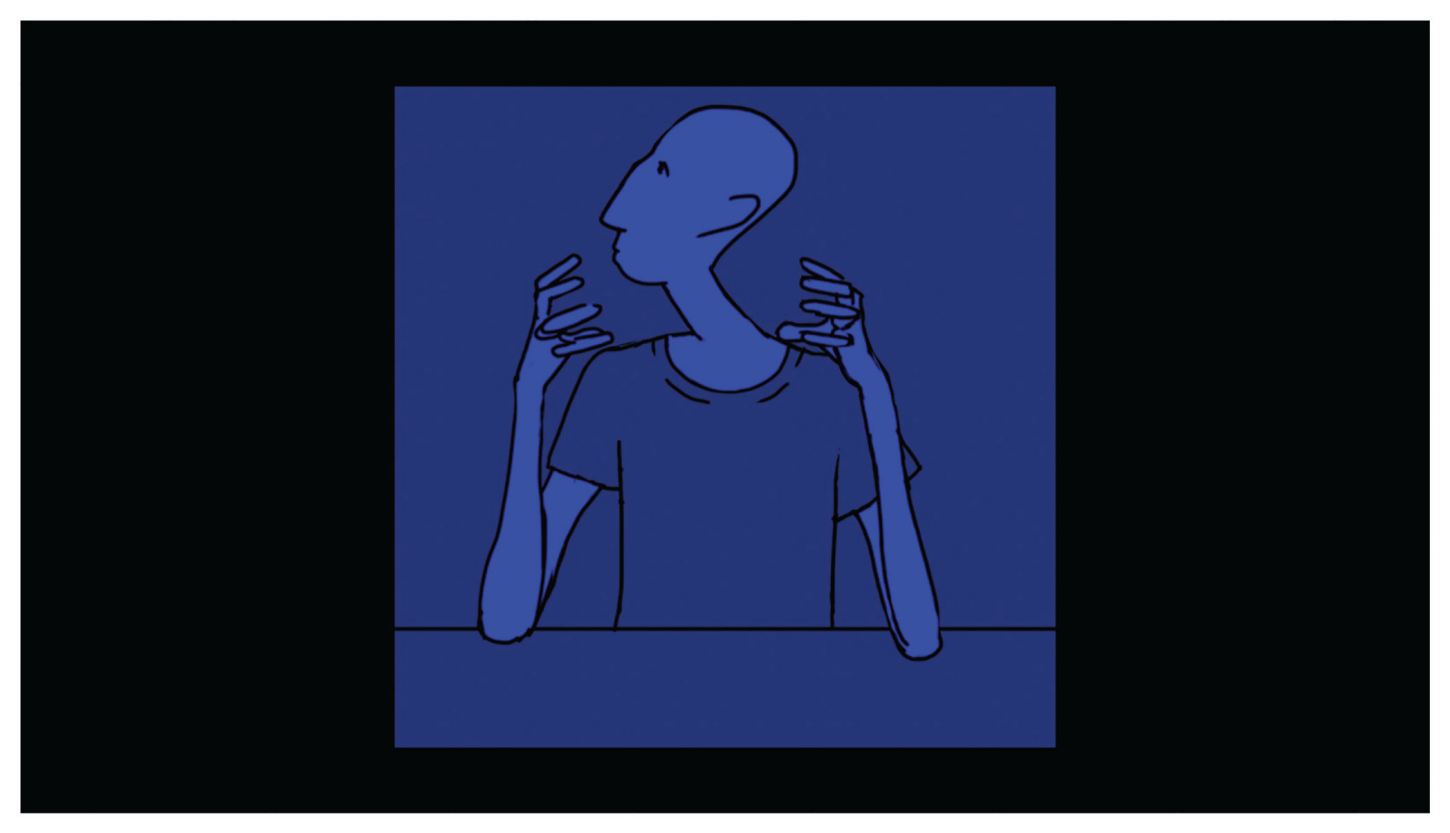
*Still from* Drawing on Autism, *A. Widdowson (dir.), 2021. UK. © Alex Widdowson*.

I directed and animated the third case study, *Drawing on Autism* (Widdowson 2021). The film was based on three interviews with a single anonymous autistic participant, a friend I had known for ten years. Following an unstructured interview about the topic of autism, I asked him if I could use a clip of his testimony for an unrelated lip sync animation exercise using a new software. When I showed this animated vignette to Professor Steven Eastwood, one of my Ph.D. supervisors, he observed that the character avatar I had created looked to him like an other-worldly alien (Figure 3), a common autism trope (Murray 2008: 98). I returned to my friend, using this vignette as the starting point for a second interview, with the goal of creating a new film about the topic of representation in animated documentary practice. When both the participant and I agreed we were not happy with my first draft of the new film’s ending, we recorded a final interview in which I asked him how he felt appearing in an animated documentary. Both the participant and I are characters in the film, our animated avatars changing from one scene to the next. The scenes of the film follow distinct aesthetic codes, some of which draw from film traditions such as science fiction and film noir, others borrowing from the aesthetics of sports illustration, medical diagrams or iconic locations such as Sigmund Freud’s couch. Each visual approach provides a meta commentary on the topics of autism representation, our power dynamics or animated documentary ethics. The influence of *A Is for Autism* is notable during scenes that depict the participant’s memories of his school years; the artwork was traced from drawings he created as a child.

## Representational risks in animated documentary practice

Employing animation to communicate documentary narratives to audiences provides a filmmaker with a set of communicative tools that significantly differ from live-action footage. When developing a framework for distinguishing between techniques employed in animated documentaries, Annabelle Honess Roe looks at what animation could do that the live-action alternative would either struggle to achieve or be incapable of (2013: 22). One of the three animated documentary functions theorized by Honess Roe is ‘non-mimetic substitution’, where animated images bear little resemblance to the physical world they represent (2013: 24). She observes that this function has allowed filmmakers to mask the identities of those appearing in documentaries for whom public scrutiny would be detrimental to their wellbeing (Honess Roe 2013: 24). This can be achieved in live-action documentaries by blurring or casting a shadow across a participant’s face, however this subtractive approach is significantly less visually stimulating than the animated alternative. Non-mimetic substitution also provides the filmmaker with the opportunity to insert expressive and/or symbolic visual material into the diegesis of the documentary, which operates like a visual metacommentary guiding the spectator’s understanding of the documentary evidence. We can see this technique employed in the three case studies. In *An Alien in the Playground*, bullies are depicted as monstrous static heads. In *A Is for Autism*, Temple Grandin is depicted as a scribbled stick figure climbing stairs when she describes her relatively delayed speech development. In *Drawing on Autism*, the participant and I are depicted as a collection of rudimentary shapes when discussing the impossibility of creating a neutral representation (see Figure 1).

**Figure 4:**
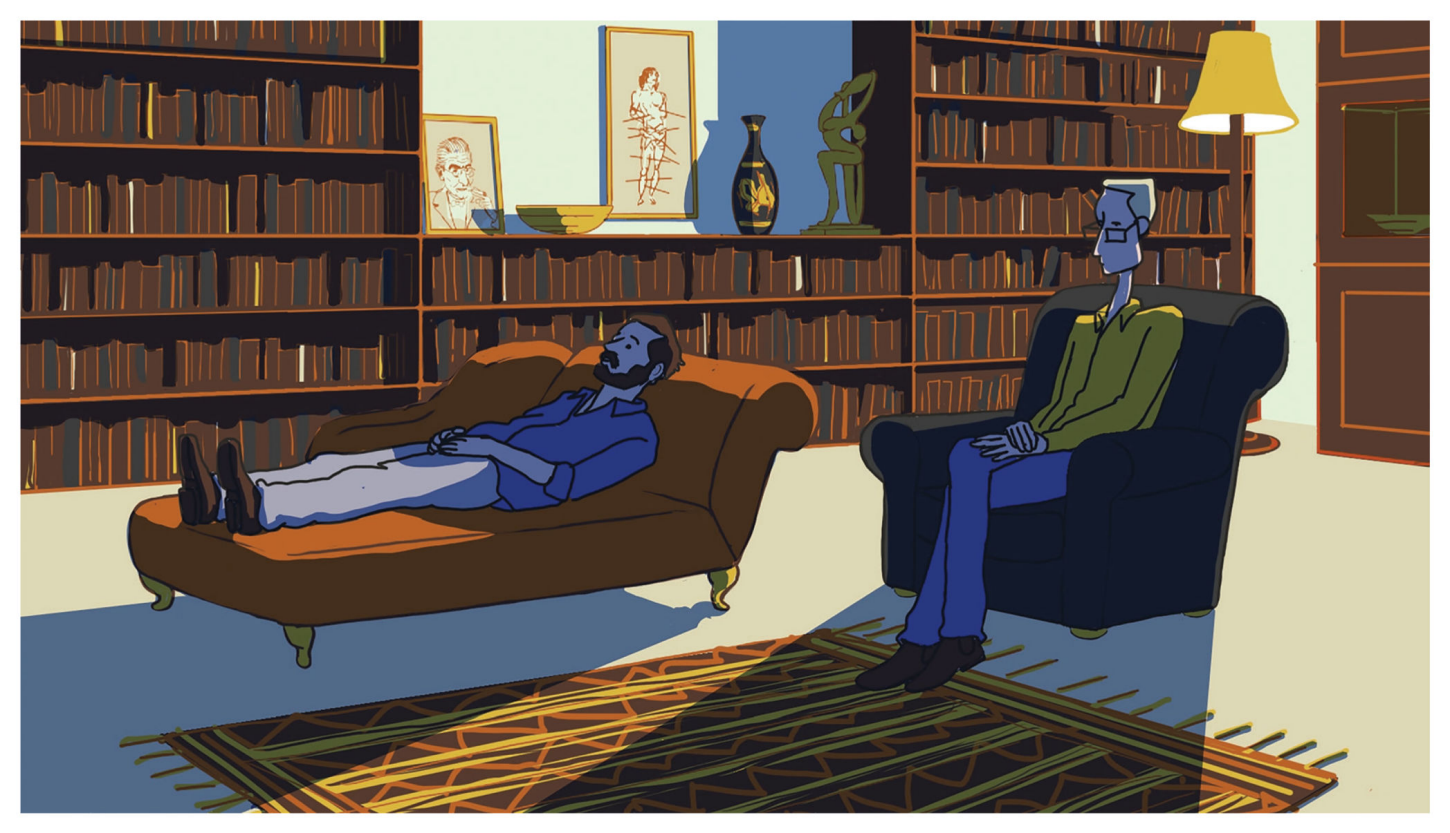
*Still from* Drawing on Autism, *A. Widdowson (dir.), 2021. UK. © Alex Widdowson*.

‘Evocation’, another of Honess Roe’s functions of animated documentary, is the attempt to visualize the cognitive processes of a documentary participant using animation (2013: 25). This has been attempted in live-action documentaries, for example, in *The Reason I Jump* (Rothwell 2021), where visually rich footage of an autistic boy exploring the land below a viaduct, connotes the optical sensory euphoria experienced by many autistics. However, the camera is a blunt tool compared to the imaginative potential of animation. The disconnection between the physical world and the artificiality of animation, what Eisenstein calls the ’plasmaticness’ of animation (Leyda and Eisenstein 1988: 21), a freedom from physical reality, allows for a vast spectrum of aesthetic possibilities when attempting to represent invisible mental phenomena. *An Alien in the Playground* features a complex web of pseudo-mathematical formula as a means to evoke Muggleton’s social strategies. *A Is for Autism* shows a collection of disconnected abstract and mimetic animated details to demonstrate the narrator’s difficulty establishing the areas in which someone else was trying to draw his attention. In *Drawing on Autism*, I depict myself lying on a chaise longue, positioning me as analysand and the participant as analyst in order to evoke my own neurotic self-scrutiny (Figure 4).

The ability of animated documentary to both mask the identity of participants and visualize mental phenomena has enabled the production of a significant number of animated documentaries featuring marginalized participants with experiences of disability, neurodivergence and/or difficulties managing their mental health. Although some of the participants in the case studies I have chosen are credited, and it appears they are each able to advocate for themselves, the wider trend adds urgency to the task of researching practice-specific ethics of animated documentary production.

Scholarship on documentary ethics provides helpful guidance for both live-action and animation practitioners, with regards to the filmmaker’s duty of care towards their documentary participants, and their responsibility to inform and empower audiences (see e.g. Ruby [1979] 2005; Pryluck [1976] 2005; Rughani 2013). However, it is important to acknowledge the need to place greater emphasis on issues of representation when discussing animated documentary ethics. There are subtle differences and overlaps in the representational risks of both non-mimetic substitution and evocation. In most live-action documentary productions, participants present a stable physical appearance that reflects their bodily presence in the world. They can determine for themselves what clothes they wear, how their bodies move and what actions they take in front of the camera. At the time of filming the participant is aware of the environment in which they appear on camera. None of this is true in interview-based animated documentaries. Animated documentary’s modular production process involves creating moving images separately from the audio interview, which are combined to form the basis for a film. In this creative process, the filmmaker has substantial representational control. As such, animated documentaries present greater representational risks when the participants have no say in the construction of the animated images. Representational risks are further exacerbated, when non-mimetic substitution and evocation are employed by the filmmaker in order to augment the interview documents with visual metacommentary, aestheticizing the topic of the film, filtered through their ideological stance and unconscious biases.

Evocation is expressed most literally when the virtual camera of an animated image can be used to adopt the point of view of a participant. However, this term can also apply to how a participant’s character avatar and surroundings are represented, when it provides insight into the participant’s cognition. As such, we start to see significant overlaps between two of Honess Roe’s functions. Whereas non-mimetic substitution is a visual metacommentary on any topic, this is referred to as evocation when a documentary participant’s cognition is the subject of visual metacommentary. This formulation is further complicated by the fact that all forms of animated metacommentary, evocative and non-mimetic, are indicative of the imagination of whomever created them. With the exception of mimetic substitutive animated documentaries, which attempt to faithfully reconstruct events from the real world that could not be filmed, we could describe most animated documentaries as evocations of the filmmaker’s imagination. Where this is the case, evocative animated documentaries, which are not autoethnographic or the product of participant collaboration, risk misleading audiences because they have the potential to function as an evocation of the film-maker’s subjective feelings about a participant, while masquerading as the participant’s cognitive perspective.

When the filmmaker and documentary participant are separate people, the filmmaker’s access to the cognitive source material is limited by the participant’s ability to translate their experiences into a communicable form, such as speech. This second-hand information must then be interpreted by the filmmaker and translated again into a visual form – evocative animation. By contrast, if the filmmaker was trying to animate their own cognitive experiences, they have unmediated access to the source material when creating the visual representation. This distinction prompted me to develop Honess Roe’s terminology in order to distinguish between three types of evocation: *first-person evocation*, where a filmmaker attempts to represent their own cognitive phenomena, *third-person evocation*, where a filmmaker attempts to represent a documentary participant’s cognitive phenomena, and *collaborative evocation*, where the filmmaker and participant work together to create an animated evocation of the participant’s cognitive phenomena. These formulations of participant/filmmaker relations are equally relevant to first-person, third-person and collaborative non-mimetic substitution. However, emphasis is shifted away from concerns about authenticity and towards the risk of generating problematic connotations.

First-person evocation is a low-risk representational practice because the filmmaker has unique authority on, and access to, their own cognitive processes. Whether the filmmaker is employing first-person evocation or non-mimetic substitution, the filmmaker/participant’s best interests, ideological stance and unconscious biases are one and the same.^4^

In third-person evocative animated documentaries, such as *An Alien in the Playground*,^5^ there are significant representational risks because there are several layers of mediation between the participant’s internal life and the animations created by the filmmaker. At the start of a third-person evocative production the filmmaker conducts research, most importantly in the form of an interview with the participant. This may even include the participant’s guidance on how to visualize their cognitive processing. However, when this information gathering is concluded, the audio edit is locked and the filmmaker develops evocative animations of the cognitive phenomena without further input or influence from the participant. Carla MacKinnon’s research into animated documentary industrial practices shows that this approach, which she calls ‘linear’, is motivated by economic prudence. Animation is a costly process so there is a powerful incentive to avoid having to revise or abandon completed animated sequences (MacKinnon 2022: 156). The filmmaker must risk putting a project over budget and/or behind schedule if, mid-way through the production, they allow a participant to suggest corrections to evocative representations of their cognitive phenomena. When a non-autistic filmmaker creates a third-person evocative animated documentary about the experience of an autistic participant, they have no way of gauging the accuracy of the depiction until it is too late for changes to be made. In the absence of the participant, the non-autistic filmmaker must draw from their own imagination and knowledge of the topic of autism which is shaped by their outsider’s perspective. As such, when the film employs third-person non-mimetic substitution and/or evocation, there may be a misalignment between the filmmaker and participant’s best interests, their ideological stances and unconscious biases. In the participant’s absence, the filmmaker’s misalignments with the participant may go unnoticed as they become embedded within the finished film.

Animated documentaries that employ collaborative evocation, such as *A Is for Autism* and *Drawing on Autism*, are constructed with considerable input from the participant(s), reducing the representational risks associated with third-party evocation. Samantha Moore’s practice-based research on improving the accuracy of evocative animated documents is an example of practice-based research into collaborative evocation (2015). Moore returned repeatedly to participants^6^ with revised versions of animated evocations of their experiences of face-blindness or phantom limb syndrome. The participants were the arbiters of when the animation was accurate enough to be considered complete (Moore 2015: 24–25). Compared to a linear third-person evocative animated documentary, Moore’s cyclical method is harder to plan and likely to require more time and funding (MacKinnon 2022: 168). However, in both evocative and non-mimetic animated documentaries, where collaboration exists during the visualization process, representational risks associated with third-party animations are mitigated. If there are misalignments between the filmmaker and participant’s best interests, their ideological stances and/or unconscious biases, the participant has the opportunity to identify them and confront the filmmaker during the course of the film’s development and/or production.

To summarize, animated documentary filmmakers take significantly different representational risks, and therefore have different responsibilities, compared to their live-action counterparts. This is true for evocation, non-mimetic substitution and where these practices overlap. As described above, these representational risks differ between first-person, third-person and collaborative evocative animated documentary practices. However, because non-mimetic substitution can also be described as an evocation of the filmmaker’s perspective on the topic of the documentary, in both evocation and non-mimetic substitution, making a film about one’s own experiences is a low-risk representational activity, representing someone else’s lived experiences presents greater risks and working collaboratively can mitigate these risks. In each of these practices, the film-maker’s best interests, ideological stance and/or unconscious biases become embedded in the construction of the film. I will now shift my attention to the means by which ideology emerges in the three case studies.

## Cinematic gaze

Gaze theory is crucial when explaining how ideology manifests in animated documentaries. Conceptions of the cinematic gaze were first developed in the 1970s by psychoanalytic film theorists, including Jean-Louis Baudry (Baudry and Williams 1974), and Christian Metz (1982), who relate the experience of cinema spectatorship to Jacques Lacan’s theorization of the *mirror stage*, an illusory encounter that starts when the child first sees themselves in the mirror (Lacan 2010). This interaction helps instigate the ego formation, an overly simplified manifestation of self-identity, that does not account for unconscious activity (McGowan 2007: 2). Baudry summarizes this approach concisely: ‘The arrangement of the different elements – projector, darkened hall, screen – in addition to reproducing in a striking way the mise-en-scène of Plato’s cave […] reconstructs the situation necessary to the release of the “mirror stage” discovered by Lacan’ (Baudry and Williams 1974: 539). From an early Lacanian perspective, these authors are arguing that the cinematic image resides in and perpetuates the *Imaginary Order*, one of three organizing realms with which humans grapple. The Imaginary Order deals with the intersection of imagery and the ego. These early Lacanian film theorists argued that cinema was an illusory force which allowed spectators to enjoy narratives by inviting them to find themselves in temporary fantasies. Through the use of psychoanalytic tools, they showed how the Imaginary Order can be lifted to reveal a hidden ideological structure, the *Symbolic Order*. In this formulation the film theorist is analyst, the spectator analysand and the film dreamlike materials to be employed in analysis (McGowan 2007: 4).

Laura Mulvey, in her seminal essay ‘Visual pleasure and narrative cinema’ (1975), developed a feminist stance based on this psychoanalytic perspective on spectatorship and the various forms of looking that take place within the cinematic experience. She argued that in classic Hollywood films there is an interplay between three sets of looks that align with the coordinates of phallocentric ideology, a synonym for patriarchy: (1) the look of the camera which is typically under the control of a male director, (2) the look of the male characters, who are typically active agents in the progression of the plot, and (3) the look of the audience from the dark comfort of the cinema, enjoying the activity of the male actors and the sexualized presentation of the female actors (Mulvey 1975: 17). Mulvey described the *male gaze* to be the phenomenon of expression and interpolation of patriarchal ideology through this three-way matrix of cinematic looking. She relied on Lacan’s theory of the mirror stage to account for female audience members misrecognizing themselves within a cinematic experience that did not serve their best interests (Mulvey 1975: 9–10). Expanding Mulvey’s conception of gaze beyond the parameters of sexism, it is the invisible, possibly unconscious, ideological stance of the filmmaker, detectable in their creative choices, embedded in a film’s narrative and aesthetics, that interpolate spectators into the filmmaker’s ideological position.

Applying gaze theory to animated documentary practice – a medium that affords the filmmaker with granular control over how the characters appear, behave and interact; a medium with no physical constraints on the way the filmmaker orchestrates and filters reality through mimesis, stylization, invention and symbolism – demonstrates that the theory is not only relevant, but that it manifests with less restraint than live action. The filmmaker’s innumerable creative interventions aestheticize documentary evidence, increasing its illusory effect on spectators. Furthermore, each of these decisions is influenced, consciously or unconsciously, by the hegemonic ideologies the filmmaker is immersed in. This process sheds further light on why third-party evocative and non-mimetic animated documentaries are high risk activities and explains how this risk increases if the filmmaker operates from a position of privilege relative to the participant. In order to explore the relationship between the animated documentary, gaze theory and autism representation, it is first necessary to review the ideological systems which have a bearing on the topic of autism.

## Autism and ideology

There is currently an ideological paradigm shift taking place in and around the autistic community. In recent years the medical paradigm for understanding autism and other forms of neurological difference has been challenged by a new framework that has largely been developed by autistic activists and academics. The neurodiversity paradigm rejects the idea that forms of neurological difference are inherently negative. Instead, they should be considered naturally occurring forms of variation in the human population. This approach is analogous to that of biodiversity, where the greater the species diversity in an ecosystem the more adaptable that system is when coping with environmental changes (Chapman 2019: 371). As such, encouraging members of a society to aspire to a norm, while marginalizing those on the periphery, weakens the aggregate health of that society. It would thus be detrimental for the human race to attempt to ‘cure’ autism (Chapman 2019: 373).

The neurodiversity paradigm also draws from the social model of disability which attempts to account for much suffering in neurodivergent communities by distinguishing between forms of difference and the disabling effects caused by lack of acceptance and accommodation from the society around them (Chapman 2019: 375). From this perspective, an autistic person with hyper-sensitivity to sound only experiences this as disabling when they enter an environment that was designed to accommodate the majority of the population, who experience lower auditory sensitivity. The neurodiversity paradigm has been primarily shaped by neurodivergent researchers who have attempted to develop an understanding of their own neurotype, drawing inspiration from first-hand insights and a shared sense of experience. For example, Damian Milton’s theory *The Double Empathy Problem* (Milton 2012) proposes that the medical theory that autistics lack cognitive empathy does not account for the situated perspective of the non-autistic researchers making this claim. Rather, the lack of empathy and understanding runs in both directions. Neurotypical people struggle to empathize with autistic modes of cognition, and vice versa.

The fact that neurotypicals and autistic people empathize effectively with members of their own neurotype shows that the medical model for understanding autism, which historically blamed autistics for this social disconnect, was and still is, to an extent, an expression of neuro-normative hegemony (Milton 2012). In recent years the authors Remi Yergeau, Athena Lynn Michaels-Dillon and Nick Walker collectively coined the term *neuroqueer*. They observed that hetero- and neuro-normative hegemony seem to operate as analogous and interconnected systems of oppression for those outside these norms (Walker 2021: 161). Walker frames neuroqueering as a verb or practice, that describes the act of embracing and/or performing one’s neurodivergence in defiance of norms, arguing for the emancipatory and creative possibilities of these acts (2021: 160–63). I will now examine how the ideological systems and tools discussed here manifest through the animated gaze in the aforementioned animated documentary case studies.

## The gaze in animated documentary

**Figure 5:**
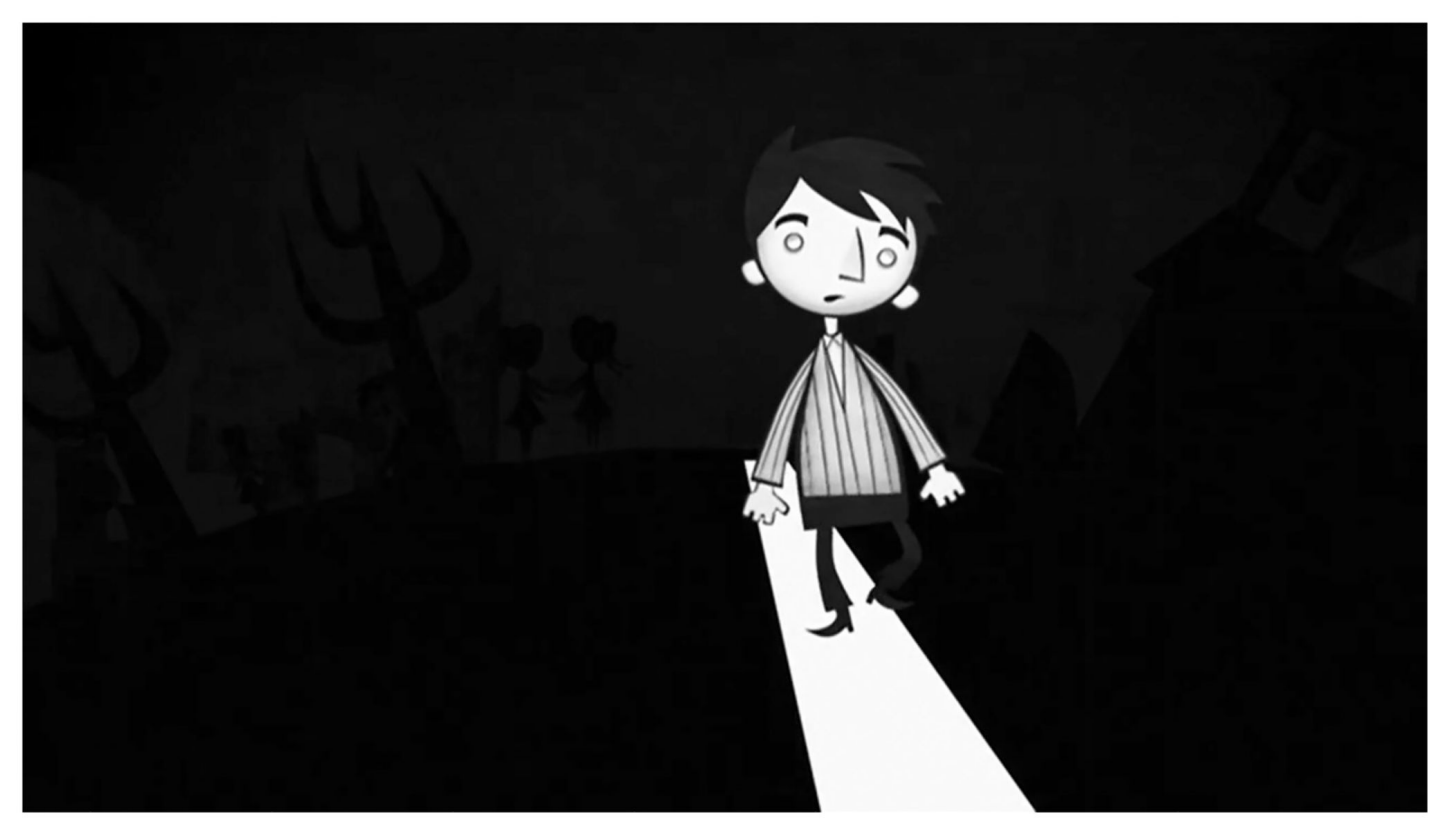
*Still from* An Alien in the Playground, A. Glynne (dir.), 2009. *UK: Channel 4. © Mosaic Films*.

*An Alien in the Playground* operates in alignment with the medical paradigm for understanding autism, its narrative structure operating like a checklist of symptoms. Glynne’s professional experience as a clinical psychologist further demonstrates the plausibility of this claim, along with the fact that the film was produced in 2009, well before the concept of neurodiversity had entered the zeitgeist. The neuro-normative medical gaze ensnares characters in a visual apparatus that frames their deviations from cognitive norms as deficiencies or deficits; little value is placed on these forms of difference. Instead, these are problems to be corrected or endured, rather than accepted and accommodated. An interesting manifestation of this attitude in the visual field of *An Alien in the Playground* is the design of Muggleton’s eyes (Figure 5), which are missing pupils. It is a common autistic trait to avoid eye contact, however this avoidance is an active response to the gaze of others, rather than a passive failure to engage (Grandin and Panek 2013: 35). Glynne’s film presents Muggleton as a boy with blank eyes, incapable of directing his gaze. This representation goes far beyond communicating an autistic distaste for staring into the glassy openings in eyeballs (Robison 2008: 3). Instead, Muggleton’s autistic gaze is erased; the inference being that, if he cannot bring himself to meet the neuro-normative gaze of the virtual camera, the other characters or the audience, then his way of looking is irrelevant.

*A Is for Autism* was made in 1992, six years before the term neurodiversity had been coined (Singer 1998: 13). However, the film taps into the contemporary trend of autistic life writing, pioneered by Temple Grandin, who appears in the film. Grandin’s books were some of the earliest publicly available sources that relied on lived experience to address the topic of autism. This first wave of autistic autobiographies was preoccupied with explaining what autism was to a neurotypical audience. Nonetheless, Grandin’s work was an early instance of autistic self-advocacy, which set a foundation on which the Neurodiversity Movement and paradigm would build. Like Grandin’s early writing, *A Is for Autism* contains some antecedent principles of the Neurodiversity Movement, such as autistic participation in research and representation, i.e. #NothingAboutUsWithoutUs.

While the film cannot be explicitly tied to this yet-to-be-formulated ideology, the choice to base each animated scene on artwork created by an autistic artist means that the surface appearance of the film is indexically bound to the imagination and creativity governed by autistic modes of expression. This strategy operates like a representational short circuit, diverting textual analyses of the film back to the labour of autistic contributors rather than the non-autistic director. Each mark made is supported by a graphic citation that links back to someone whose claim of authenticity is unimpeachable. While this is true of the drawn imagery, the same cannot be said for the film’s syntax; Webb maintains control of the narrative flow and editing. The film thus harmonizes the self-advocating autistic gaze with that of an ally; a way of looking that suggests admiration and affection for the art. According to Webb, before the idea of a collaboration was proposed to him, his motivation for making a film about autism was his appreciation for the work of the autistic artist Stephen Wilshire (Webb 2021). The fetishization of autistic art is present throughout the film, a mode of fascination that echoes Jean Dubuffet’s *Art Brut* movement. Looming behind the celebratory surface of this film, gazing back at us, is the hidden puzzle piece icon; the preferred visual metaphor of 90’s autism charities, which positions a child’s autism as a mystery that if solved would reveal the authentic self. While *An Alien in the Playground* exemplifies the medical gaze, *A Is for Autism* reflects the beginnings of an ideological shift. Despite being rooted in the fetishizing gaze of an ‘outside art’ collector, the way this film fore-grounds autistic ways of looking indicates the antecedent elements of the neurodiversity paradigm.

*Drawing on Autism* was produced as my understanding of the neurodiversity paradigm developed. While I had not yet been indoctrinated when starting the film’s development, by the time it was complete, my ideological beliefs and self-understanding had aligned with the neurodiversity paradigm, further augmented by neuroqueer theory. During the second interview, which makes up the body of the film, I was deeply concerned with my ability to speak about autism as someone not diagnosed autistic. Furthermore, I had started to develop concerns that third-party evocative animated documentary was at best misleading. As such, I attempted to manifest these anxieties in the production design and offer visual metacommentary on the director/participant relationship. Each scene contextualizes our power dynamics or foregrounds the mechanisms of representation by placing us in a new thematically linked scenario.

I think it is interesting that I fail to mention in the film my own neurodivergence; I am schizoaffective. Looking back, I can see this is an invaluable framework through which I can deconstruct how my gaze manifests in this film. As someone who, in their early 20s, experienced a total implosion of identity through psychosis, and who still experiences tidal shifts in affect, my world-view has been shaped by this initial trauma and my untethered disposition. I have a perennial preoccupation with maintaining heightened self-awareness, as demonstrated by my self-scrutiny in the film. I am constantly attempting to articulate and bolster my sense of self, best illustrated by my choice to appear as a character in my last three animated documentaries, while consistently depicting myself in the same clothes. Having once been extraordinarily unreliable, I have compensated by actively trying to prove myself to be trustworthy in each aspect of my life. During this production, I offered my participant the power to veto scenes they did not like, suggest changes or pull out entirely, all the way through the development of the film. Giving the participant full oversight of the film meant that without their trust the film would never have been made. As such, despite not being declared, the film is organized by my own neurodivergent gaze.

My failure to acknowledge my neurodivergent positionality in the text puts into question the possibility of reading this film as an act of neuroqueering. Furthermore, the overcompensatory behaviour I enact indicates that I am not yet at peace with my difference and still strive after the acceptance of a neurotypical society. However, the constantly changing aesthetic and dense symbolism in each environment of the film indirectly celebrates schizoaffectivity: steadily migrating along an affective spectrum makes me feel I have seen the world through a variety of lenses, and I remember the early stages of psychosis feeling like the opening of semiotic floodgates. It is also worth acknowledging that the dialogue was recorded before my neuroqueer gaze manifested in animated images. This correlates with my gradual adoption of the neurodiversity paradigm and neuroqueer theory.

A further way this film could be seen as an act of neuroqueering is the way the medium itself parallels the slipperiness and performativity of my own understanding of identity, including gender and sexuality. Inspired by Judith Butler’s analysis of drag queens (1990: 186–89) – where biological sex, gender and performance collide – I gained a better understanding of how animation operates in a documentary context when I thought of the hybrid practice as *documentary drag*. Animation omits the truth claims associated with indexical imagery, a helpful analogy for how drag severs connections to biological sex.^7^ In the place of photography, animation imitates the performative codes and conventions of the live-action documentary genre, the aesthetic of realism which is rehearsed and standardized throughout the documentary canon (Bruzzi 2006: 217). Documentary conventions are a helpful analogy for gender performance, the socially determined signifying practices that regulate conceptions of men and women. If we swap the words ‘gender’ and ‘drag’ for ‘documentary’ and ‘animation’ in the following Butler quote, we can see how animated documentary operates as a post-structuralist challenge to documentary tradition: ‘In imitating gender [documentary], drag [animation] implicitly reveals the imitative structure of gender [documentary] itself’ (Bulter 1990: 187, emphasis added).

Where appropriate, animation’s unique capacity for plasmaticness (Leyda and Eisenstein 1988: 21) and visual excess (Honess Roe 2013: 44) are employed in a documentary context. Here, animation disrupts and subverts documentary conventions; similarly, drag performances both exaggerate and embellish gender signifiers. Christina Formenti argues that animated documentary should be catalogued separately from documentary proper, labelling it as ‘the sincerest form of docudrama’ (2022: 46). I feel more excited by the creative potential of lifting these partitions, using animation to continue queering the documentary genre. This attitude, enacted in *Drawing on Autism*’s form, combined with the content that actively celebrates neurodiversity and the aesthetic manifestations of my schizoaffectivity, demonstrate the film’s employment of the neuroqueer gaze.

The fact I was not conscious of this in the early stages of the film’s production, going so far as to cut out the reference the participant made to my diagnosis, demonstrates the role that unconscious activity had in this example of practice. I will now look closer at unconscious bias and parapraxis to develop this insight.

## Animated documentary and the unconscious

Bill Nichols famously equated documentary practice with the ‘discourses of sobriety’ (1991: 3), disciplines ‘seldom receptive to”make-believe”[…] sobering because they regard their relation to the real as direct, immediate, transparent’ (1991: 3–4). Nichols’s characterization is contrasted by Elizabeth Cowie, who argues there is tension in all documentary practice between the ‘scientific recording of what one sees and somehow the desire to give it meaning and perhaps make it more beautiful’ (Piotrowska 2012: 90). She refers to these as ‘contradictory desires’ (Cowie 2011: 2). Cowie also identifies unconscious desires present in the makeup of documentary productions, shifting the nature of the debate from a discourse of sobriety to a ‘discourse of desire’, in which the director is pursuing and delivering pleasure as well as knowledge to their spectators (1999: 25). Michael Renov extends this argument calling documentary a ‘discourse of jouissance’, the Lacanian term for transgressive enjoyment, suggesting the filmmaker’s unconscious desires are likely to be exercised through the practice amidst attempts to represent reality (2004: 23). Agnieszka Piotrowska goes further by drawing upon psychoanalysis to understand the dynamics between documentary filmmaker and participant (2012). She asserts that the psychoanalytic relationship is a useful metaphor for understanding the power dynamics and intimacy involved in documentary productions featuring human participants (Piotrowska 2012: 54).

Interestingly, Nichols makes a similar claim by referring to Michael Foucault’s analysis of the asymmetrical power relations that occur in regulated systems of interpersonal exchange (Foucault 1990: 65). Nichols includes the documentary interview, along with Foucault’s reference to the psychoanalytic therapeutic relationship, as examples of power relations whose origins can be traced back to the medieval Christian tradition of confession (2017: 146). Crucially, Piotrowska differentiates the documentary relationship from that of the therapeutic, due to the stark contrast between the confidentiality of one practice and the public spectacle of the other:

The point is not that the documentary encounter is ‘like’ psychotherapy or psychoanalysis; it is rather the exact opposite: through the structure of the encounter and powerful unconscious mechanisms a situation might arise leading to a profound ‘misrecognition’ on the part of the subject of the film and the filmmaker alike. A documentary encounter might feel like a special safe place in which one is listened to and even loved, but that private space will soon enough be turned into a public spectacle – a process which carries with it inherent dangers.

(2012: 56)

Piotrowska’s thesis centres on the role played by transferential love in the documentary participant/filmmaker relationship, facilitated by the asymmetrical power balance. However, her observations, along with those of Renov and Cowie’s, illuminate the risk of unconscious activity affecting documentary production. The foundational insight of psychoanalysis, developed by Sigmund Freud, was that a human’s self-awareness as a unified subject is an illusion; we are in fact divided, constituted from both conscious and unconscious elements. The Freudian unconscious is a repository of needs and desires, wishes and memories that influence behaviour despite not being accessible to the conscious psyche (Thurschwell 2000: 4).

Freud developed the concept of *parapraxis*, commonly known as *Freudian slips*, to explain the revelatory properties of many accidental actions and utterances. He argued that closer analysis of errors, such as forgetting something important and mispronunciation of words, provide invaluable glimpses into unconscious truths (Thurschwell 2000: 34). Parapraxis can help further explain the assertion that third-party evocative and non-mimetic animated documentaries present higher representational risks. First-person and collaborative animated documentaries are just as likely as their third-person counterparts, to have parapraxes enter the audio testimony, narrative structure or animated aesthetics. However, mistakes made by a filmmaker representing their own lived experiences are merely providing astute spectators the opportunity to infer deeper insights into the film-maker’s unconscious feelings about the topic of discussion. Because the filmmaker has an insider perspective of the topic, the risks of these errors, no matter how undermining, at worse reflect internalized prejudice and thus reveal meaningful insights into the real-world impact of living as some-one affected by the topic of the film. Parapraxis presents less of a threat to collaborative filmmakers, because the participant is on hand to sense check their work. If an unintended connotation is noticed by the participant, the filmmaker is afforded the opportunity to remove it from, or address it in, the film. This was the case in *Drawing on Autism*, where I brought to the attention of the participant the possibility that I mistakenly represented them as an alien. However, instead of agreeing, the participant questioned my approach, pointing out that I was attempting to employ a metatextual method to extend my control over the narrative of the film.

A third-person filmmaker, tasked with inventing animated interpretations of the lived experience of an absent participant, is in a similar position to the first-person filmmaker, with no supervisory presence to curtail their creative freedom. However, if parapraxes emerge, in the form of unintendedly problematic connotations, these slip-ups reflect the filmmaker’s privileged naivety, an outsider who is not directly impacted by the topic of the documentary. The title of the film, *An Alien in the Playground*, is to me an interesting example of parapraxis. It draws attention to the bullying experienced by Muggleton, but nowhere in his narration does he mention the autism-alien stereotype. If this comparison was not brought up by Muggleton in the long form version of the interview, then Glynne’s choice of title is problematic, because he characterizes Muggleton in a way that echoes the abuse. There is not enough context provided by the film to know for sure if Glynne is disrespecting his participant ironically, an act that perhaps reveals unconscious hostility, or, alternatively, Glynne consciously thought an extra-terrestrial alien would be an appropriate way to frame Muggleton’s neurological difference. In which case, Glynne is further exposing his unconscious alignment with neuro-normative hegemony. Both options seem misaligned with the honourable intentions detectable throughout Glynne’s work.

The reason parapraxis presents a threat to documentary participants and the communities affiliated with the topics addressed in documentaries is because of the ubiquity of unconscious bias among humans. Unconscious biases are the behaviours and beliefs we perform or possess in relation to an *Other*. Nobody operates in the world without bias. Our conscious understanding of self is formulated in the conscious and unconscious relations to the various iterations of the Other we experience during our development. Each of us have ‘blind spots’ in regard to our own biases; we cannot always detect our stigmatizing behaviours or attitudes (Crosby 2019: 23–24).

## Stereotyping and Othering

The study of prejudice was developed across a broad spectrum of humanities and social sciences research. Stereotyping was a related concept developed from the 1950s onwards, used to explain how beliefs about social categories of people developed and operated, associated with types of behaviour, personality traits and motivations (Dervin 2012: 186). Together with stereotyping, the verb ‘Othering’ is another invaluable concept when discussing animated documentary filmmaker/participant relationships. The sociological definition of Othering is the process of establishing an essential difference between the Self and the Other in a way that suggests the latter’s inferiority (Krumer-Nevo and Sidi 2012: 300). Michal Krumer-Nevo and Mirit Sidi have researched unintentional Othering of participants in ethnographic studies. Ethnography, like much of documentary practice, involves in-depth interviews with people about their personal experiences, before the material is analysed, interpreted and presented publicly. The shared heritage of documentary and ethnographic practices, their similar qualitative methods and comparable epistemic goals, as well as the emphasis placed on issues of representation in Krumer-Nevo and Sidi’s research, indicates that animated documentary studies will benefit from these ethnographic insights on when Othering occurs and how it can be addressed.

Krumer-Nevo and Sidi study the mechanisms by which marginalized research participants can be Othered in ethnographic writing by researchers who occupy a relative position of privilege. They establish four processes, each of which increases the risk of a participant being Othered: *objectification, decontextualization, dehistoricization* and *deauthorization* (Krumer-Nevo and Sidi 2012: 300).

*Objectification* involves the exclusion of an individual’s specific complexity and personal perspective, reducing their representation to that of a stereotype (Krumer-Nevo and Sidi 2012: 300). While it is common for animated documentaries to focus on the complexities of a participant’s subjectivity and lived experience, the risk of objectification is raised when non-mimetic substitution is used to anonymize a participant. Removing the representational features of a participant risks shifting their presentation of identity away from the particular, while leaving in place visual clues that link them to the social categories they are affiliated with. When symbolic or expressive embellishments are employed, they provide the filmmaker an opportunity to visually express or symbolize their unconscious attitude towards those categories, by drawing on, for instance, problematic stereotypes. This was precisely the mechanism that led me to depict my autistic friend as a blue man with an elongated neck in the clip that instigated the production of *Drawing on Autism*.

*Decontextualization* is where behaviours are identified in isolation from the context and motives that led to their development, rendering reasoning as irrational (Krumer-Nevo and Sidi 2012: 300). Anthropomorphic character designs have been employed in numerous animated documentaries, helping the filmmaker avoid directly representing visible characteristics of race, religious affiliation, gender, etc. Through this, stereotypical caricaturing can potentially be avoided, but simultaneously it trivializes the participant’s humanity and removes them from the intersecting systems of power and oppression that have influenced their subjective experience of the world (e.g. *It’s Like That* [Southern Ladies Animation Group 2003]).

**Figure 6:**
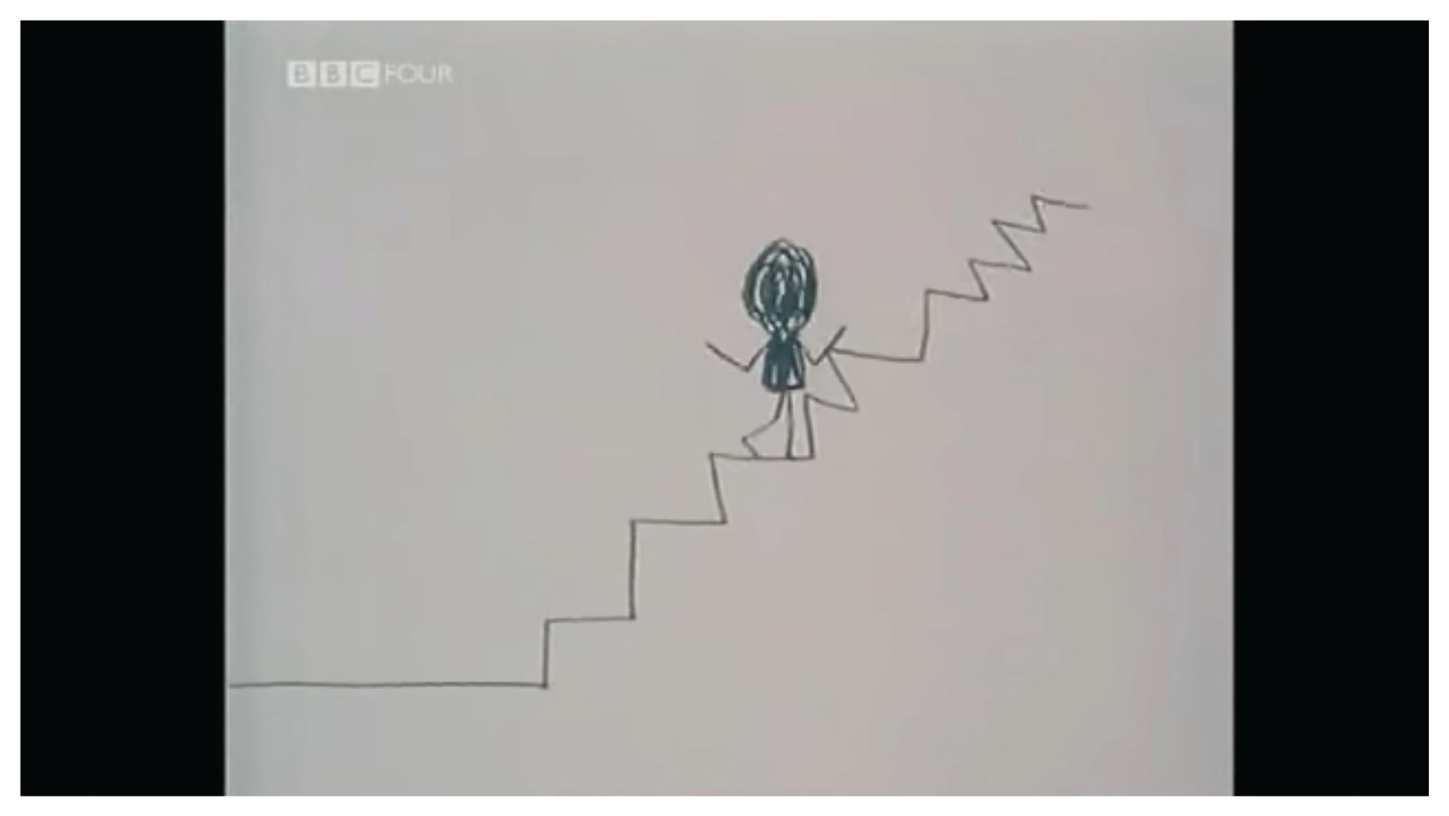
*Still from* A Is for Autism, T. Webb (dir.), 1992. *UK: Channel 4. © Channel 4*.

*Dehistoricization* indicates an over-emphasis on present circumstances, ignoring the implications of an individual’s history (Krumer-Nevo and Sidi 2012: 300). Animated documentary is an extremely labour-intensive mode of production. As such, there is an incentive to make these films as concise as possible. In my own practice, a film that is between five and ten minutes long will likely originate from two to six hours of interview material. This ratio demonstrates how much contextualizing information must be excluded in order for me to progress effectively using this form. Despite being the shortest of the case studies, Glynne does a good job of chronicling Muggleton’s struggles across primary and secondary school in *An Alien in the Playground*. In stark contrast, in *A Is for Autism*, the contributions of seven participants are split into short, isolated segments. One would not know from the film that Grandin is decades older than the other participants and grew up amidst an entirely different autism paradigm, a psychoanalytic framework that causally linked autism to so-called ‘refrigerator mothers’ (Silberman 2015: 187). All aspects of her adult life are omitted, including her ground-breaking autism research and activism. Instead, the film presents a handful of her memories from childhood. This bolsters the popular misconception that autism is a childhood condition, a stereotype that reflects the anxieties and influence exerted by parents of autistic children (Murray 2008: 142). The additional juxtaposition of her testimony with another autistic artist’s childlike drawings is particularly infantilizing (Figure 6), considering the precision she employs in her professional practice as a world-leading industrial designer.

*Deauthorization* is where a representation of the participant appears as an omniscient, objective record, rather than a product of the selective interpretation of an author (Krumer-Nevo and Sidi 2012: 300). The disconnection between audio testimony and animated image allows for greater flexibility when editing the audio. This, combined with the sub-genre’s tendency towards personal narratives, contributes to the frequency in which the interviewer’s questions are removed, as is the case in *An Alien in the Playground* and *A Is for Autism*. This is referred to as the ‘masked interview’, which allows a participant to appear to be narrating their own story, rather than presenting the conversation between filmmaker and participant in which that information was recorded (Nichols 2017: 136). The masked interview is particularly problematic in third-party evocative animated documentaries. In *An Alien in the Playground*, Glynne is able to present his own visual fantasies of what it must be like to be autistic, while disguising the appropriation by removing himself from the dialogue. Muggleton’s first-person narration then appears alongside these evocative animations as if to corroborate their authenticity, despite him not being involved in their creation.

## Methods for reducing representational risks in animated documentaries

In response to their findings, Krumer-Nevo and Sidi developed three modes for resisting Othering in ethnographic writing, each of which are easily applicable to animated documentary practice: *narrative, dialog* and *reflexivity*.

*Narrative* presents the participant as a subjective agent in their story, contextually influenced by their community, environment and era (Krumer-Nevo and Sidi 2012: 301–02). As previously demonstrated, the brevity of animated documentaries, their tendency towards masking identities and emphasis on contemporary interviews, each contributes to the risk of objectification, decontextualization and dehistoricization of participants. Krumer-Nevo and Sidi’s conception of narrative should prompt animated documentary filmmakers to consider these three risks, when planning interviews, building the audio edit and designing non-mimetic character avatars.

*Dialog* requires interpretations of participant testimony be subjected to secondary critique, by either allowing the participant to offer feedback, attempt the task independently or by the researcher presenting their primary interpretations alongside other possible readings (Krumer-Nevo and Sidi 2012: 302–03). In the context of animated documentary, Krumer-Nevo and Sidi’s first iteration of dialog could be equated with *collaborative evocation* and *collaborative non-mimetic substitution*, when the filmmaker and participant discuss each stage of the film’s construction. This corroborates my earlier observations about how collaboration can reduce representational risks associated with thirdparty animated documentary practices. Collaboration was the first foundational principle I adopted when creating *Drawing on Autism*. The second iteration of dialog is comparable with the participant being afforded the opportunity to animate parts of the film and could be described as *participant led production*, a practice that is exemplified by Daniel Sellers’s train sequences in *A Is for Autism*. The third mode of dialog, best described as *self-critique*, was the second foundational principle adopted in the production of *Drawing on Autism*. This approach prompts the filmmaker to, first, consider the limitations of their own subjective perspective. Second, they should examine how these perspectives are enmeshed in an ideological framework and consider whether this ideology works against the best interests of the participant. It can also be valuable to investigate, and maybe even adopt, the participant’s own ideological stance, as I did in regard to the neurodiversity paradigm. Third, the filmmaker should be open to the possibility that they possess unconscious biases, as do all people. They should attempt to develop self-awareness, consider the connotations of parapraxis if it occurs and foster the kind of documentary relationship that would allow the participant to raise any concerns.

*Reflexivity* requires the audience be provided with the necessary information to decode an author’s method and approach. *Positional reflexivity* involves exposing to the reader the author’s presence, their history and position to reveal their analytical subjectivity and bias (Krumer-Nevo and Sidi 2012: 305–06). This resembles Nichols’s ‘participatory mode’ of documentary, where the filmmaker’s interactions with participants are included in the film (2017: 137–38). Positional reflexivity was the third foundational principal I adopted in *Drawing on Autism*. My presence in the film encourages audiences to connect the imaginative content of the animation to my own subjectivity, rather than falsely interpreting it as evocations of the participant’s autistic subjectivity. However, as previously mentioned, I believe I should have been more transparent about my own neurodivergence. It would have also helped audiences to contextualize the participant’s candour and playfulness by explaining in the film that we had already been friends for ten years.

*Textual reflexivity*, the fourth foundational principle I adopted in *Drawing on Autism*, requires the otherwise hidden or unnoticed mechanisms of production and manipulation of source material be exposed to the audience (Krumer-Nevo and Sidi 2012: 305–06). Sybil DelGaudio argues that animated documentary always functions as its own textual ‘metacommentary’, because the non-indexical medium prompts audiences to analyse its capacity to inscribe actuality (1997: 192). However, I believe that it is useful to explicitly emphasize this tendency in animated documentary practice, by employing techniques comparable to the Brechtian *Verfremdungseffekt* (Weber and Heinen 1980: 32). Laura Mulvey draws a similar conclusion when co-directing her own film, *The Riddle of the Sphinx*, with Peter Wollen (1977). She uses textual reflexivity to fracture the spectator’s illusory connection to the cinematic apparatus and raise their awareness about the ideological structures imbued in the symbolic order of cinema. In *Drawing on Autism*, I steered the topics of discussion to a number of textually reflexive themes, including the ongoing construction of the film, the representational risks associated with third-party non-mimetic substitution, the fallacy of third-party evocation and the problematization of the documentary truth claim. I also employed aesthetic strategies to promote textual reflexivity in the film. These included: (1) deliberately depicting production equipment, such as microphones, to remind people of the atypical nature of the conversation; (2) disrupting temporal and physical space by dissolving the boundaries of the recording of the interview and historical context of re-enacted scenes, e.g. the participant is depicted as a child while his character avatar’s lip sync matches his contemporary account of childhood memories; (3) shifting between illustrative styles and character designs to discourage illusions of representational reliability. In this ten-minute film there are at least twelve distinct aesthetic strategies. Lastly, (4) designing the environment of the scenes in response to the topics of discussion. Shifting between these locations drew attention to the modular disconnect between image and voice, further illuminating animation’s role as visual metacommentary.

## Conclusion

In this article I have used as case studies *An Alien in the Playground, A Is for Autism* and my own film, *Drawing on Autism*, to explore the ways in which documentary ethics shift towards issues of representation in animated documentary practice. I have illuminated how third-party non-mimetic substitution and evocation present particularly high representational risks. I analysed how these risks manifest through the cinematic gaze, exposing the filmmakers’ positions within the contemporary ideological power structures, some of which contributed to the marginalization of autistic participants. I have demonstrated how parapraxis can expose a filmmaker’s unconscious biases about autism, and I have also looked at how stereotypes and Othering representations further marginalize autistics in these animated documentaries.

It is worth noting that while I make many critical observations about the film *An Alien in the Playground*, these are made through the lens of the contemporary neurodiversity paradigm. Andy Glynne unashamedly draws from his experience as a clinical psychologist and may well still believe the medical paradigm has the potential to help autistics. Perhaps more importantly, it would appear that Joshua Muggleton also leans towards the medical paradigm. He is now a qualified psychologist, who, on his website (mugsy.org), describes himself as ‘a person with autism’, rather than an autistic person, and refers to autism spectrum ‘disorder’, rather than condition (Muggleton n.d.), both of which have been campaigned against by the Autism Self Advocacy Network, a leading organization in the Neurodiversity Movement (autisticadvocacy.org). Glynne and Muggleton seem to be in ideological alignment, which seriously undermines arguments that this film worked against the participant’s best interests. However, their position contradicts the preferences and demands of autistic led advocacy organizations, by promoting a perspective of autism that frames autistic difference as a problem to be solved.

*A Is for Autism* has received near-universal praise, so much so that the film is referred to as ‘a classic’ of the animated documentary genre (Webb 2021). Despite my affection for this film, it has been productive to delve deeper into the layers of signification embedded in the text, some of which have problematic connotations. That said, I find it astonishing how closely aligned the film is with the cutting edge of early 90’s autism self-advocacy, and how it contains many antecedent elements of the neurodiversity paradigm.

When drawing influence from these two films, along with the theoretical considerations detailed in this article, I established four guiding principles that helped me create *Drawing on Autism*: (1) collaborative evocation and collaborative non-mimetic substitution, (2) self-critique, (3) positional reflexivity and (4) textual reflexivity. These tools were the means by which I conducted the experiment of creating an animated documentary that puts ethics at the forefront of its conception. However, these principles should not be considered a normative code to be followed slavishly. They are the result of context-specific research by practice. Rather, I am hopeful these theoretical tools may help other filmmakers to enrich their own practice-based research into ethical animated documentary production techniques.

## Footnotes

1 In this article I will uniformly refer to humans who are not the filmmaker, appearing in documentaries as ‘participants’. There are other terms for this role, including documentary subjects, however for the sake of consistency I will be using the terminology established in BillNichols’s *Introduction to Documentary* (2017: 31).

2 Muggleton also states on his own website that he prefers person first language. I am a strong advocate for identity first language, but in this instance, I will adhere to his preferences.

3 The *Animated Minds* website refers to Muggleton’s diagnosis as Asperger’s syndrome (animatedminds. com). Following the publication of *DSM-V* in 2013, Asperger’s syndrome is no longer an active diagnostic category because it meets the diagnostic criteria for autism spectrum disorder (Miller 2021). Autistic self-advocates prefer the synonym autism spectrum condition, which holds fewer negative connotations (asiam.ie).

4 Examples of first-person evocative animated documentaries on the topic of autism include *Cloud Boy: An Autistic Journey* (Firth-Bernard 2021), *HOLE* (Goletski 2018) and *My Thoughts on Autism… and Orangutans* (Minchin 2022).

5 Glynne states in an interview that participants had no say over their character design or the storyboard, and the visualization process was not discussed with them. They did, however, sign off the audio edit of their interview (Glynne 2012: 297–98).

6 Moore referred to participants as collaborative consultants (2015: 71).

7 It is also worth noting that the ontological and epistemic foundations of both biological sex and documentary’s evidentiary basis have been problematized in recent decades (see Butler 1990, on biological sex and Trinh T. Minh-Ha 1990; Bruzzi 2006, on documentary truth claims).
